# Proximity co-evaporation growth of SnSe thin films for high-responsivity photodetectors

**DOI:** 10.1039/d5ra08877a

**Published:** 2026-02-06

**Authors:** Sailakshmi Janga, Kurapati Kalyan, Shaik M. Abzal, Arshad Ahamed A, Manve Rasik Ramesh, Rajkumar Patel, Jatis Kumar Dash

**Affiliations:** a Department of Physics, SRM University-AP Amaravati 522240 India jatis.d@srmap.edu.in jatiskumar@gmail.com; b Energy & Environmental Science and Engineering (EESE), Integrated Science and Engineering Division (ISED), Underwood International College, Yonsei University 85 Songdogwahak-ro, Yeonsugu Incheon 21983 South Korea rajkumar@yonsei.ac.kr

## Abstract

In this work, tin selenide (SnSe) thin films were successfully synthesized using a cost-effective proximity co-evaporation method within a chemical vapor deposition (CVD) system. The process involved a thermally evaporated Sn thin film and selenium powder as precursors, arranged in a source-substrate-face-to-face configuration to enable uniform and self-limiting lateral growth under an inert atmosphere. X-ray diffraction (XRD) confirmed the formation of orthorhombic SnSe with high crystallinity and phase purity. Field emission scanning electron microscopy (FESEM) and energy-dispersive X-ray spectroscopy (EDS) revealed a uniform grain morphology and near-stoichiometric composition, while X-ray photoelectron spectroscopy (XPS) confirmed the presence of Sn^2+^ and Se^2−^ oxidation states. Optical studies revealed a band gap of ∼1.15 eV, aligning well with the ideal range for optoelectronic applications. Electrical measurements demonstrated ohmic contact behavior, and photoresponse analysis under white LED illumination exhibited a significant enhancement in photocurrent, with a responsivity of 29.9 A W^−1^, detectivity of 3.8 × 10^11^ Jones, and quantum efficiency of 67.45%. These results show that the fabricated SnSe thin films could be suitable candidates for high-performance photodetectors and optoelectronic devices.

## Abstract 2

In this work, tin selenide (SnSe) thin films were successfully synthesized using a cost-effective proximity co-evaporation method within a chemical vapor deposition (CVD) system.

## Graphical abstract

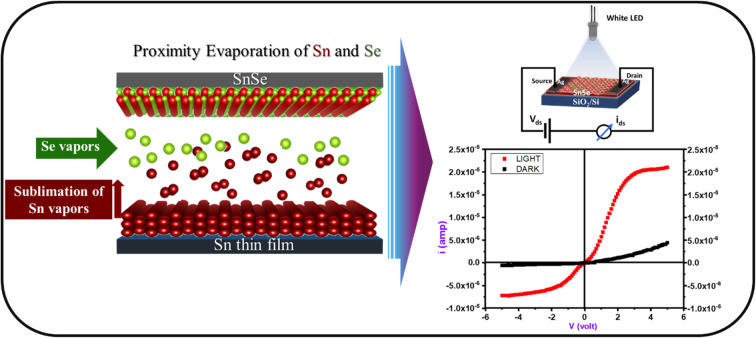

## 1. Introduction

The emergence of two-dimensional (2D) atomic crystals has opened new frontiers in materials science, owing to their remarkable physical properties that differ significantly from their bulk counterparts.^1,2^ This has led to an expanding field of research into a variety of 2D systems, including layered metal dichalcogenides,^3–5^ metal oxides,^6,7^ and MXenes,^8,9^ each offering unique dimension-dependent physical and chemical characteristics.^2,10–13^ These materials have a wide range of applications, particularly in optoelectronics,^14,15^ electronics,^16,17^ sensing,^18,19^ and energy storage devices.^20,21^ Among the emerging 2D systems, Group IV–VI monochalcogenides such as MX (*e.g.*, SnS, SnSe, SnTe) and MX_2_ (*e.g.*, GeS_2_, SnS_2_, SnSe_2_)^22^ exhibit favorable electronic and photoelectronic properties.^23–25^ SnSe and SnSe_2_, in particular, are narrow-bandgap semiconductors with high carrier mobilities of 10^4^ cm^2^ V^−1^ s ^−1^ and 233 cm^2^ V^−1^ s ^−1^ respectively.^26^ The bandgap of SnSe spans from a direct transition at ∼0.9 eV to an indirect one at ∼1.3 eV.^27–29^ Due to its tunable band gap,^29^ material abundance, and low toxicity,^30^ SnSe is an attractive candidate for optoelectronics^31,32^ and photovoltaic devices.^27,33^ Also, its band gap falls within the optimum range for solar cell applications.^34,35^

SnSe has a layered orthorhombic arrangement of atoms, with lattice parameters *a* = 11.49 Å, *b* = 4.44 Å, and *c* = 4.135 Å, which is similar to a bi-planar layered structure.^35–37^ This structure can be thought of as a distorted version of a rock-salt (NaCl) structure.^38^ The bulk SnSe exhibits a layered orthorhombic structure that belongs to the *Pnma* space group. Its anisotropic behaviour can be observed when viewed from different directions, such as along the *a*, *b*, and *c*-axial directions. Several synthesis methods have been developed to fabricate SnSe, ranging from solution-based routes like solvothermal,^39,40^ hot injection,^41^ one-pot,^41^ and surfactant-free synthesis,^42^ to vapor-phase techniques such as physical vapor deposition (PVD) based thermal evaporation,^43^ inert gas condensation,^44^ flash evaporation,^45^ hot wall epitaxy, molecular beam epitaxy,^46,47^ sputtering,^48^ and chemical vapor deposition (CVD) techniques.^49,50^ While many of these methods can yield high-quality films or nanostructures, they often suffer from drawbacks such as high temperature requirements, toxic reagents, limited scalability, poor control over stoichiometry, and significant equipment and fabrication costs. To overcome these challenges, we employ a proximity-assisted co-evaporation technique integrated within a CVD system for the controlled growth of thin SnSe films. In this approach, a thermally evaporated Sn thin film and selenium powder are used as simple elemental precursors, and the growth substrate is placed in close proximity to the Sn source, resulting in a short diffusion path for the reacting species. This configuration enables efficient and localized interaction between Sn vapor and Se species, promotes self-limited vertical growth, and improves stoichiometric uniformity while minimizing secondary phase formation. Unlike conventional CVD or PVD processes that require precise precursor flow control, high vacuum, or hazardous chemicals, the present method operates under an inert atmosphere using readily available precursors, making it cost-effective, reproducible, and scalable. These features make the proximity-assisted co-evaporation approach particularly attractive for the synthesis of high-quality SnSe thin films for optoelectronic applications.

## 2. Experimental details

The SnSe films were grown by utilizing a physical vapor deposition (PVD) method to deposit Sn thin film and a chemical vapor deposition (CVD) technique, utilizing Se powders & Sn thin film as precursors, to co-evaporate the materials.

### 2.1 Thin film deposition using PVD

**Fig. 1:**
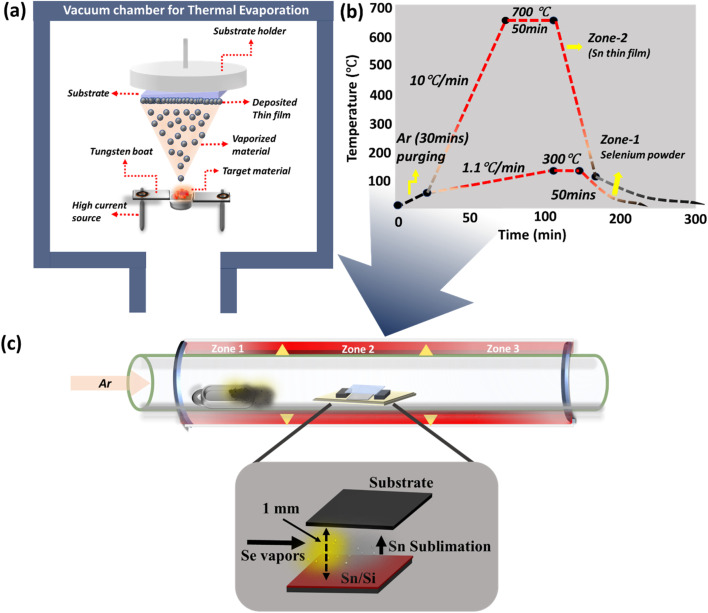
(a) Schematic of the thermal evaporation-based PVD setup used for depositing Sn thin films onto Si substrates. (b) Temperature profile applied during the CVD co-evaporation process, showing Se source at 300 °C and Sn precursor zone at 700 °C with a 50-minute dwell time. (c) Schematic diagram of the proximity-assisted CVD setup for growing SnSe thin films, illustrating the placement of Se powder and Sn/Si film along the quartz tube with a ∼1 mm gap between the precursor and target substrate.

A 50 nm Sn thin film was initially deposited onto a cleaned Si (100) substrate using a thermal evaporation-based physical vapor deposition (PVD) method. Tin granules (99.9%) obtained from Loba Chemie Pvt. Ltd, served as the source material, which were placed in a tungsten (W) boat inside the high-vacuum chamber (base pressure ∼7 × 10^−6^ mbar). The deposition rate was maintained at 0.6 Å s^−1^, monitored using a quartz crystal microbalance (QCM) and the source-to-substrate distance was fixed at 15 cm. A schematic of the thermal evaporation setup is depicted in Fig. 1(a).

### 2.2 Proximity co-evaporation inside CVD furnace

SnSe thin films were synthesized *via* proximity evaporation in a controlled chemical vapor deposition (CVD) system. The setup consisted of a 1-meter-long quartz tube housed in a three-zone temperature-controlled furnace equipped with a programmable Eurotherm 3508 PID controller.

A 50 nm thick Sn thin film, pre-deposited on Si (100) substrate by thermal evaporation, was used as the Sn precursor and placed in the central zone (zone-2) of the furnace. The source material selenium (Se) powder (99.9%) was purchased from Loba Chemie Pvt. Ltd A clean glass substrate was mounted face-down over the Sn film with a ∼1 mm gap, achieved by inserting two quartz spacers (∼1 mm thick) at the edges of the quartz plate (Fig. 1(c)).

This face-to-face proximity setup promoted self-limiting lateral growth of SnSe thin films. Se powder (700 mg) was loaded into a quartz boat and placed in zone-1 of the furnace. The entire system was purged with high-purity argon gas (99.99%) at 2000 sccm for 20 minutes to eliminate residual air and moisture. During growth, the flow rate was decreased to 1000 sccm to maintain an inert atmosphere. The Se powder in zone-1 was heated to 300 °C, while the Sn/Si precursor stack in zone-2 was heated to 700 °C and maintained (dwell time) for 50 minutes. This temperature profile facilitated the efficient evaporation of Se and its diffusion across the 1 mm gap, where it reacted with the sublimated Sn, leading to the growth of SnSe thin films on the substrate. After the completion of growth, the furnace was allowed to cool naturally to room temperature over approximately 2 hours under continuous Ar flow, preventing oxidation or contamination. The short gap configuration (∼1 mm) facilitated uniform, SnSe film growth with self-limited vertical stacking and improved stoichiometry. A schematic of the temperature profile and CVD setup used for the SnSe growth are shown in Fig. 1(b) and (c).

### 2.3 Characterization techniques

To obtain comprehensive insights into the properties of the as-grown SnSe thin films, a range of characterization techniques was employed to examine their crystal structure, surface morphology, optical absorption, and electrical performance. X-ray diffraction (XRD) analysis was carried out using an EMPYREAN SERIES 3 diffractometer equipped with Cu Kα radiation (*λ* = 1.5405 Å) to determine the crystal structure, phase purity, and preferred orientation. The scans were performed with a step size of 0.013° and a scan time of 45 seconds per step. Surface morphology and grain distribution were analyzed using field emission scanning electron microscopy (FESEM), while elemental composition and stoichiometry were confirmed through energy-dispersive X-ray spectroscopy (EDS) coupled to the SEM system. Atomic force microscopy (AFM) was used to measure the thickness of the SnSe films. To investigate the chemical states of Sn and Se, X-ray photoelectron spectroscopy (XPS) was carried out, focusing on the Sn 3d and Se 3d core-level spectra. The optical absorption properties were studied using a Shimadzu UV-2600i spectrophotometer in transmission mode over the wavelength range of 300–800 nm, with background correction from a bare quartz substrate. Electrical measurements and photoresponse behavior, were performed at room temperature using a Keithley 2636 B source meter. Photocurrent measurements were carried out using silver two-probe contacts spaced 1 mm apart, with illumination provided by a white LED source spanning 550 nm. A voltage sweep ranging from −6 V to +6 V was applied using gold probe tips connected to the Keithley source meter. All measurements were conducted at room temperature under ambient atmospheric conditions.

## 3. Results & discussion

### 3.1 X-ray diffraction: structural analysis

**Fig. 2:**
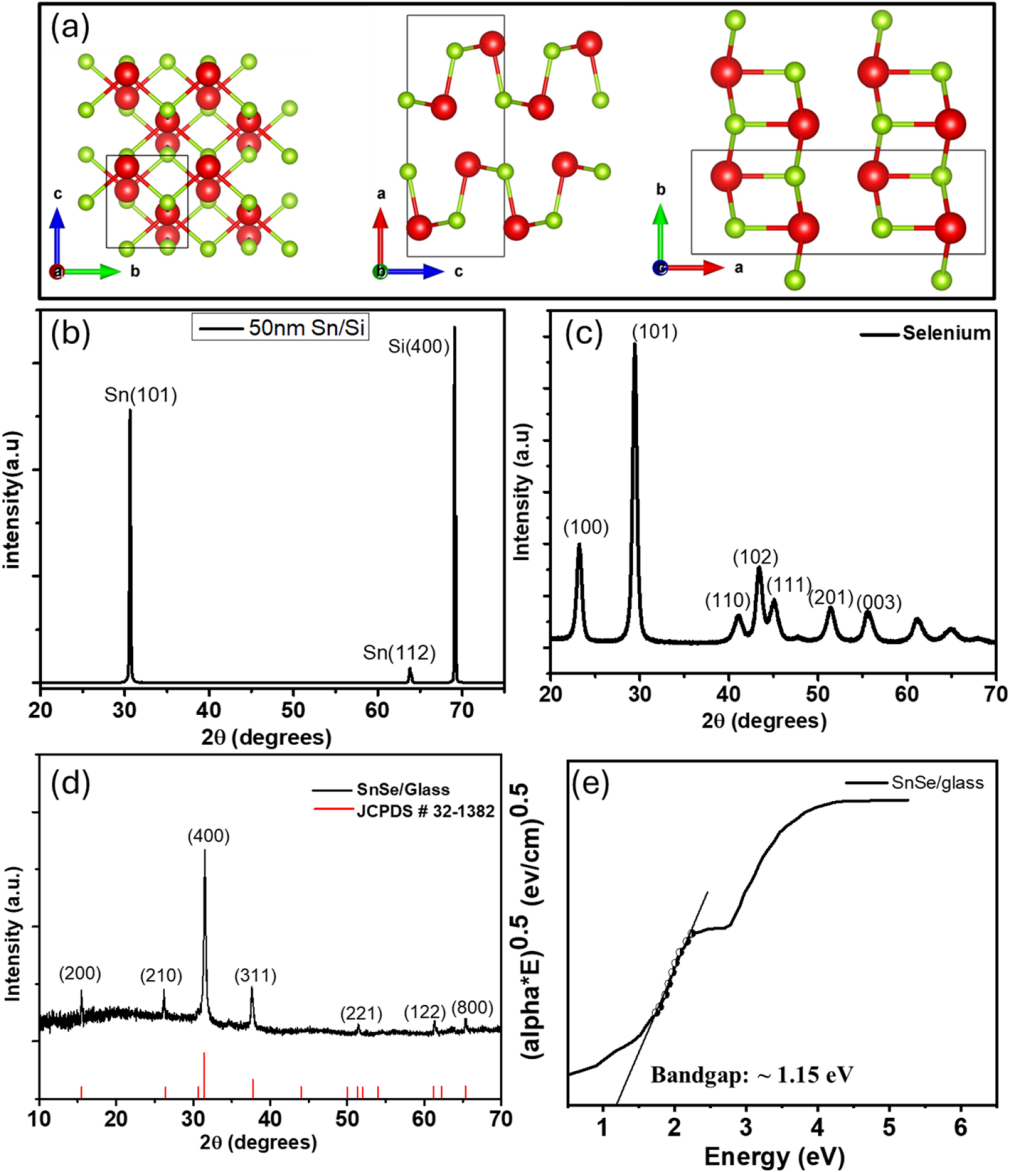
(a) Crystallographic projections of the orthorhombic SnSe unit cell along the *a*-axis, *b*-axis, and *c*-axis showing layered atomic arrangements with Sn (red) and Se (green) atoms. (b–d) XRD patterns of Sn/Si thin film, Se powder and SnSe thin film grown on Glass substrate (e) Tauc plot used to estimate the optical band gap (∼1.15 eV) from UV-vis absorption spectra.

Tin selenide (SnSe) exhibits a layered orthorhombic crystal structure belonging to the *Pnma* space group, which results in anisotropic properties along the crystallographic axes, as shown in Fig. 2(a). When viewed along the *a*-axis, the structure appears as slabs, with two layers thick and strong Sn–Se bonds in the b and c plane (plane of slabs) and weak bonds along the *a*-axis. The projection along the *b*-axis takes on a zigzag accordion-like shape, while along the *c*-axis it displays an armchair-like projection.^51,52^ The unit cell of SnSe is made up of eight atoms arranged in two adjacent layers perpendicular to the longest axis.^53^ Each tin atom forms six heteropolar bonds: three nearest-neighbor bonds within the same double layer, two next-nearest neighbor bonds in the same layer, and one bond connecting to a neighboring double layer. These bonds together form a distorted octahedron around each Sn atom. This anisotropic bonding structure influences both the electrical and optical properties of SnSe. The phase and crystallinity of the SnSe thin films were confirmed by X-ray diffraction (XRD) analysis performed at room temperature. The XRD pattern of the as-grown SnSe film, shown in Fig. 2(d), exhibits well-defined peaks indexed to the (200), (210), (400), (311), (221), (122), and (800) planes, with the (400) reflection being most prominent. These peaks are in excellent agreement with the orthorhombic phase of SnSe, as per (JCPDS Card No. 32-1382, (orthorhombic SnSe, *Pnma*)), and consistent with orthorhombic SnSe reported in the literature.^54–56^

No additional peaks corresponding to unreacted tin or selenium are detected, confirming the phase purity of the SnSe films. For comparison, the XRD patterns of thermally evaporated Sn/Si film (Fig. 2(b)) and selenium powder (Fig. 2(c)) show distinct peaks that are absent in the final SnSe film.

To evaluate the microstructural quality of the SnSe films, crystallite size, dislocation density, and strain were calculated. From the XRD data, the average crystallite size (*D*_C_) was calculated using the Scherrer formula, *D*_C_ = *Kλ*/(*β* cos *θ*), where *K* is the shape factor (∼0.9), *λ* is the wavelength (1.5405 Å), *β* is the full width at half maximum (FWHM) of the diffraction peak in radians, and *θ* is the Bragg angle. The dislocation density (*δ*) was determined as *δ* = 1/(*D*_C_)^2^, and the microstrain (*ε*) was estimated using the relation *ε* = *β*/(4 tan *θ*). These calculations were summarized in Table 1. When averaged across all measured planes, the SnSe thin film exhibits a crystallite size of 42.41 nm, a dislocation density of 6.30 × 10^14^ lines per m^2^, and a microstrain of 0.0029, indicating a highly crystalline and well-ordered structure.

**Table 1:** Calculated structural characteristics of SnSe thin films from XRD data

SnSe (*hkl*) orientations	Crystalline sizes (*D*_C_) (in nm)	Dislocation density (*δ* × 10^14^ lines m^−2^)	Strain (*ε*)
(200)	40.12	6.22	0.0064
(210)	38.83	6.63	0.0039
(400)	63.94	2.44	0.0020
(311)	34.96	8.18	0.0030
(221)	33.84	8.73	0.0023
(122)	35.63	7.87	0.0018
(800)	49.61	4.06	0.0012
Average	42.41	6.30	0.0029

### 3.2 Optical band gap

The absorption capabilities of the synthesized SnSe thin film were studied using UV-vis absorption spectra. The optical band gap was obtained using the equation (*αhν*)^*n*^ = *A*(*hν* − *E*_g_), where α is the absorption coefficient, *hν* is is the photon energy, *A* is a wavelength-independent constant, *n* is the type of transition (*n* = 1/2 in this case for indirect bandgap), and *E*_g_ is the optical energy band gap. A Tauc plot was constructed by plotting (*αhϑ*)^1/2^*vs. E* (*h*ν), as shown in Fig. 2(e). The linear portion of the Tauc plot was extrapolated to intercept the photon energy axis at (*αhϑ*)^1/2^ = 0 and the estimated optical band gap of the SnSe thin film is ∼1.15 eV. This value falls within the typical range reported for SnSe thin films and nanostructures, which aligns well with the reported values in previous studies ^27–29^.

To investigate the influence of synthesis temperature on the optical properties, UV-vis absorption measurements were carried out for SnSe thin films grown at 300 °C, 400 °C, 500 °C, and 600 °C. The corresponding Tauc plots are provided in the SI (Fig. S1). The extracted optical band gaps show a clear temperature-dependent trend, decreasing from 1.9 eV (300 °C) to 1.8 eV (400 °C), 1.4 eV (500 °C), and 1.2 eV (600 °C). This progressive band-gap narrowing with increasing synthesis temperature is attributed to the formation of phase-pure SnSe and reduced structural disorder at higher temperatures.

### 3.3 Scanning electron microscopy and atomic force microscopy analysis

**Fig. 3:**
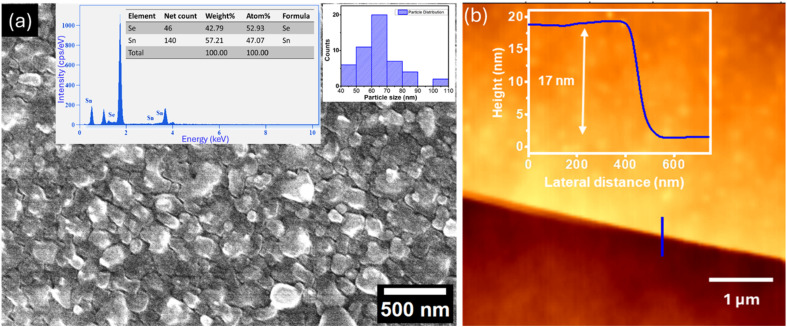
(a) FESEM image of the SnSe thin film showing uniform granular morphology; the inset displays the corresponding EDS spectrum confirming near-stoichiometric Sn and Se composition, along with the particle size distribution histogram. (b) AFM topography image of the SnSe thin film with the corresponding height profile measured across the film edge, revealing a thickness of approximately 17 nm.

The surface morphology of the as-grown SnSe thin films was examined using FESEM. The FESEM images, shown in Fig. 3(a), reveal that the film surface is composed of densely packed grains with relatively uniform distribution. The inset in Fig. 3(a) displays the grain size distribution histogram, from which the average grain size was estimated to be ∼70 nm. This grain size reflects the aggregation of smaller crystallites into larger clusters during the growth process. To investigate the elemental composition and verify the stoichiometry of the thin film, energy-dispersive X-ray spectroscopy (EDS) was carried out in conjunction with the SEM imaging. The EDS spectrum is presented in the inset of Fig. 3(a), with the inset showing the quantitative atomic percentages of tin (Sn) and selenium (Se). The analysis confirms the presence of both Sn and Se in near-stoichiometric proportions, consistent with the expected composition of SnSe. The absence of detectable impurity peaks in the EDS spectrum suggests high purity of the grown film. The uniform grain morphology observed in FESEM, combined with stoichiometric elemental composition verified by EDS, indicates successful synthesis of high-quality SnSe thin films using the proximity evaporation-assisted CVD method.

To determine the film thickness and validate the thin-film nature of the synthesized SnSe, atomic force microscopy (AFM) measurements were performed. Fig. 3(b) shows a representative AFM height profile acquired across the film edge. The corresponding line-scan analysis reveals a thickness of approximately 17 nm, confirming the formation of a thin SnSe film.

To further investigate the influence of synthesis temperature on surface morphology, SEM analysis was performed on SnSe thin films grown at different temperatures (300 °C, 400 °C, 500 °C, and 600 °C). The corresponding SEM micrographs are provided in SI (Fig. S2). A clear temperature-dependent evolution of morphology is observed, with finer grains at lower temperatures and progressively larger, more densely packed grains at higher temperatures. This trend is attributed to enhanced surface diffusion and thermally activated grain growth at elevated temperatures.

### 3.4 Elemental composition analysis

**Fig. 4:**
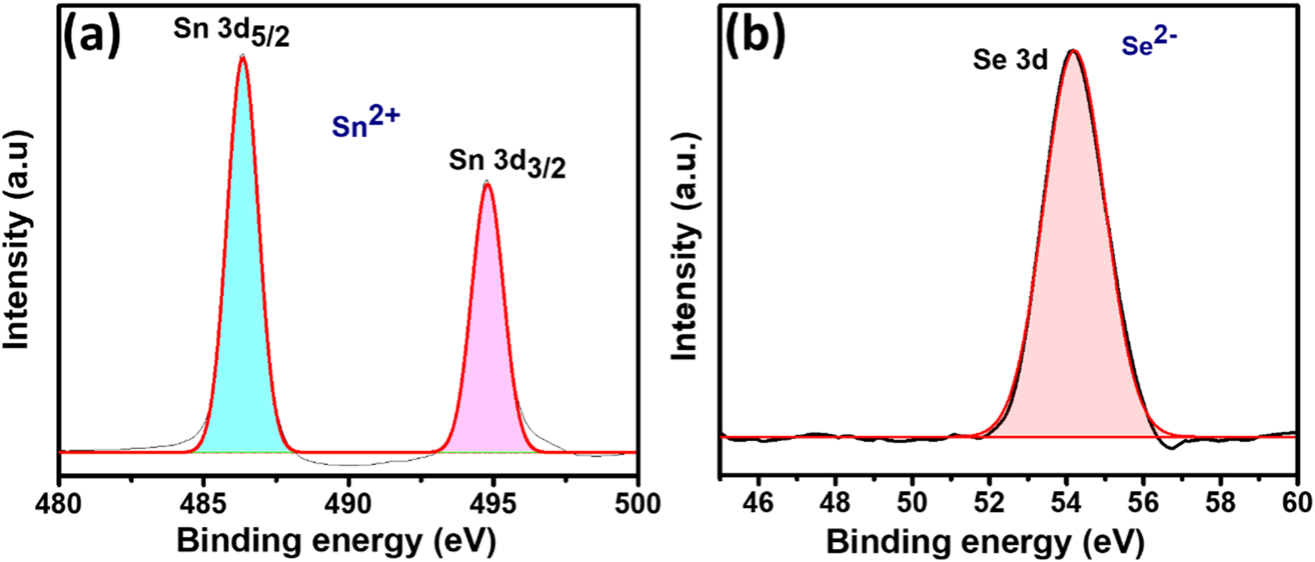
High-resolution XPS spectra of the as-grown SnSe thin film: (a) Sn 3d core-level spectrum showing peaks at 486.36 eV (Sn 3d_5/2_) and 494.82 eV (Sn 3d_3/2_), consistent with Sn^2+^ oxidation state in SnSe. (b) Se 3d spectrum exhibiting a peak at ∼54.2 eV, indicating the Se^2−^ state. These results confirm the chemical bonding environment in SnSe.

The chemical composition and electronic states of the elements present in the as-grown SnSe thin films were analyzed using X-ray photoelectron spectroscopy (XPS). High-resolution spectra were obtained for the core levels of Sn 3d and Se 3d, as shown in Fig. 4(a) and (b), respectively.

The Sn 3d spectrum in Fig. 4(a) exhibits two well-defined peaks at binding energies of 486.36 eV and 494.82 eV, corresponding to the Sn 3d_5/2_ and Sn 3d_3/2_ states, respectively. The energy separation between these peaks is approximately 8.46 eV, which is characteristic of the Sn^2+^ oxidation state in SnSe.^49,57,58^ Similarly, the high-resolution Se 3d spectrum [Fig. 4(b)] shows a prominent peak at around 54.2 eV, corresponding to the Se 3d core level, which indicates the presence of Se^2−^ states in SnSe. The binding energy values for both Sn and Se are consistent with previously reported XPS studies of stoichiometric SnSe thin films and nanostructures.^49,57,58^

### 3.5 Electrical properties

#### 3.5.1 Current–voltage measurements

**Fig. 5:**
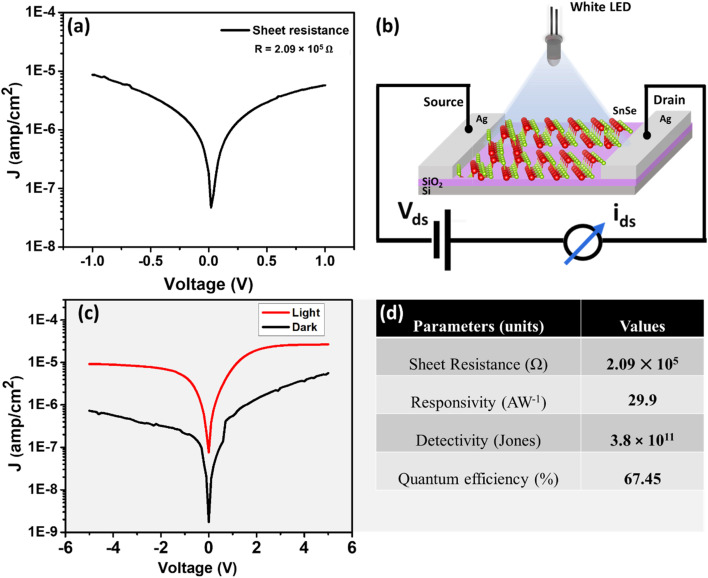
(a) *I*–*V* characteristics of the SnSe thin film under ambient conditions, enabling calculation of sheet resistance. (b) Schematic representation of the two-probe photoresponse measurement setup under white LED illumination (550 nm). (c) *I*–*V* curves under dark and illuminated conditions showing enhanced current under light exposure due to photogenerated carriers. (d) Summary of extracted photodetector parameters including responsivity, detectivity, and quantum efficiency.

To evaluate the electrical transport characteristics of the SnSe thin films, current–voltage (*I*–*V*) measurements were carried out under ambient conditions. The *I*–*V* curve for the as-grown SnSe thin film is shown in Fig. 5(a), indicating good ohmic contact between the metal electrodes and the SnSe film.

The silver electrodes used in the measurement have a work function in the range of 4.26–4.73 eV, which closely matches that of SnSe. This alignment facilitates efficient charge carrier injection and separation, enabling low contact resistance and effective extraction of photogenerated carriers. The *I*–*V* behavior shown in Fig. 5(a) further confirms that the device exhibits negligible Schottky barrier formation, which is desirable for photodetector applications.

From the slope of the *I*–*V* curve, the resistance (*R*) of the SnSe thin film was calculated using Ohm's law, *V* = *IR*. The inverse of the slope yields *R* = 2.09 × 10^5^ Ω. This resistance can be attributed to the polycrystalline nature of the film, where grain boundaries and surface roughness introduce scattering sites that impede charge transport. The charge transport behavior is also influenced by the film's microstructure. The granular surface observed in FESEM and the nanocrystalline grain size confirmed by XRD may introduce additional scattering centers. However, the film still supports stable and measurable current conduction under applied bias. The *I*–*V* results confirm that the SnSe thin films possess sufficient electrical conductivity and good electrode contact quality, forming a reliable platform for optoelectronic applications such as photodetectors and photovoltaic devices.

#### 3.5.2 Photoresponse measurements

To evaluate the optoelectronic performance of the SnSe thin film, photoresponse measurements were performed under white LED illumination (with a peak wavelength of 550 nm). The experimental setup is illustrated schematically in Fig. 5(b). The *I*–*V* characteristics of the device were recorded both in the dark and under illumination, as shown in Fig. 5(c).

Under illumination, the device exhibited a substantial enhancement in current compared to the dark state, indicating strong photoconductive behavior. The resistance of the SnSe film in the dark was measured to be 1.03 × 10^6^ Ω, whereas under white LED illumination, the resistance dropped significantly to 1.20 × 10^5^ Ω. This decrease in resistance under illumination is attributed to the generation of electron–hole pairs when incident photons with energy equal to or greater than the band gap are absorbed. The resulting photocarriers contribute to increased conductivity by participating in charge transport across the film.

The photoresponse of the device was quantified using key optoelectronic performance parameters such as responsivity (*R*_*λ*_), detectivity (D*) & quantum efficiency (QE). Mathematically, responsivity is defined as the ratio of the output photocurrent to the incident light power density per unit area. A higher responsivity indicates that the photodetector generates a larger current for a given amount of incident light, making it more sensitive to light.^63^ It is defined as 
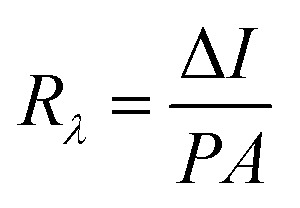
, where Δ*I* represents the difference between the photocurrent generated under illumination and the dark current, *P* is the power density of the light source, and *A* is the area of the sample exposed to the light illumination 1 cm^2^.

The detectivity (D*) is another critical performance parameter, which is defined as the ability to detect optical signals. It is represented as 
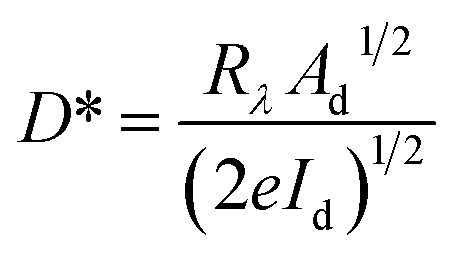
, where *A*_d_ is the effective area of the optical sensor, *I*_d_ is the experimental dark current, and *e* is the electronic charge.

Quantum efficiency (QE) is another key performance parameter for characterizing optical sensors. It is defined as the ratio of the number of charge carriers collected by the photodetector to the number of photons of a given energy incident on the optical sensor.^63^ It is calculated by using the formula 
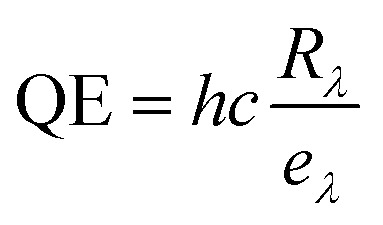
, where *h* is Planck's constant *λ* is the exciting wavelength, *c* is the speed of light, and *e* is the electronic charge.

Using these equations, the responsivity, detectivity, and quantum efficiency of the SnSe thin film under 550 nm light illumination were calculated to be 29.9 A W^−1^, 3.8 × 10^11^ Jones, and 67.45%, respectively. These values signify efficient photocarrier generation and extraction in the SnSe film. The high performance is likely attributed to a photoconductive gain mechanism ^64^, where photogenerated holes are trapped at defect sites, allowing electrons to circulate multiple times before recombination. This leads to an amplified photocurrent and consequently high responsivity.^64^

To assess the device performance, Table 2 presents a comparative analysis of the present SnSe photodetector with previously reported devices based on SnSe thin films or bulk crystals under various illumination conditions.^59–62^ The responsivity and detectivity achieved in this study are comparable to, or better than, those reported previously, highlighting the effectiveness of the proximity-assisted CVD growth method in producing high-quality SnSe thin films for advanced optoelectronic applications.

**Table 2:** SnSe thin film-based photodetector comparison with other works

Material	Morphology	Incident light (nm)	Dark current (amp)	Photodetector performance			
				Responsivity (A W^−1^)	Detectivity (jones)	QE (%)	References
SnSe	Thin film	10 600	2 × 10^−5^	0.16	3.9 × 10^7^	Not reported	59
SnSe	Thin film	404	28.3 × 10^−6^	277.3	7.6 × 10^11^	Not reported	60
SnSe	Thin film	370–808	Not reported	5.5	6.0 × 10^10^	Not reported	61
SnSe	Bulk	760	Not reported	0.003	8 × 10^7^	Not reported	62
SnSe	Thin film	550	1.92 × 10^−6^	29.9	3.8 × 10^11^	67.45	This work

## 4. Conclusion

SnSe thin films were successfully synthesized using a proximity-assisted chemical vapor deposition (CVD) method, employing thermally evaporated Sn films and selenium powder as precursors. X-ray diffraction analysis confirmed the formation of orthorhombic SnSe with a dominant (400) peak. Surface morphology observed *via* FESEM showed a uniform granular texture with an average grain size of ∼70 nm, while EDS and XPS analyses confirmed the stoichiometric presence of Sn^2+^ and Se^2−^ ions, indicating the formation of a chemically phase-pure film. Optical measurements revealed a band gap of ∼1.15 eV, aligning well with previously reported values and suggesting the film's suitability for visible to near-infrared optoelectronic applications. Electrical *I*–*V* measurements exhibited ohmic behavior, between the electrodes and the SnSe film. Under white light illumination, the device showed a significant photoresponse, with a calculated responsivity of 29.9 A W^−1^, detectivity of 3.8 × 10^11^ Jones, and a quantum efficiency of 67.45%. These results confirm that the synthesized SnSe thin films exhibit promising optoelectronic properties, making them suitable candidates for applications in photodetectors, sensors, and next-generation photovoltaic devices.

## Author contributions

J. K. D. and R. P. jointly conceptualized and supervised the project. S. J. performed the thin film synthesis. K. K., S. M. A., A. A. and M. R. R. contributed to material characterization, including X. R. D., F. E. S. E. M., UV-vis, and electrical measurements. Data analysis and interpretation were carried out by S. J. and K. K. under the guidance of J. K. D. The manuscript was written by S. J. and K. K. with inputs from all authors. All authors reviewed and approved the final version of the manuscript.

## Conflicts of interest

The authors declare that they have no known competing financial interests or personal relationships that could have appeared to influence the work reported in this paper.

## Supplementary Material

RA-016-D5RA08877A-s001

## Data Availability

Data will be made available on reasonable request. Supplementary information (SI): Tauc plots and FESEM micrographs for the SnSe thin films grown at different temperatures. See DOI: https://doi.org/10.1039/d5ra08877a.
