# Supplementary material for: Proximity co-evaporation growth of SnSe thin films for high-responsivity photodetectors
Source: RSC Adv. 2026 Feb 6;16(9):7854–62. doi: 10.1039/d5ra08877a (PMC12879990; doi:10.1039/d5ra08877a)
Supplement: RA-016-D5RA08877A-s001 [file RA-016-D5RA08877A-s001.pdf]

Supplementary Information (SI) for RSC Advances.
This journal is © The Royal Society of Chemistry 2026

Supplementary information for:

Proximity Co-evaporation Growth of SnSe Thin Films for High-Responsivity Photodetectors

Sailakshmi Janga [a,#], Kurapati Kalyan [a,#], Shaik M. Abzal [a], Arshad Ahamed A [a], Manve Rasik Ramesh [a], Rajkumar Patel [b,*], and Jatis Kumar Dash [a,*]

[a]*Department of Physics, SRM University-AP, Amaravati 522240, India*

[b] *Energy & Environmental Science and Engineering (EESE), Integrated Science and Engineering Division (ISED), Underwood International College, Yonsei University, 85 Songdogwahak-ro, Yeonsugu, Incheon, 21983, South Korea.*

# These authors have equally contributed to this work.

*Corresponding Authors

Jatis Kumar Dash, Email: jatis.d@srmap.edu.in, jatiskumar@gmail.com

Rajkumar Patel, Email: rajkumar@yonsei.ac.kr

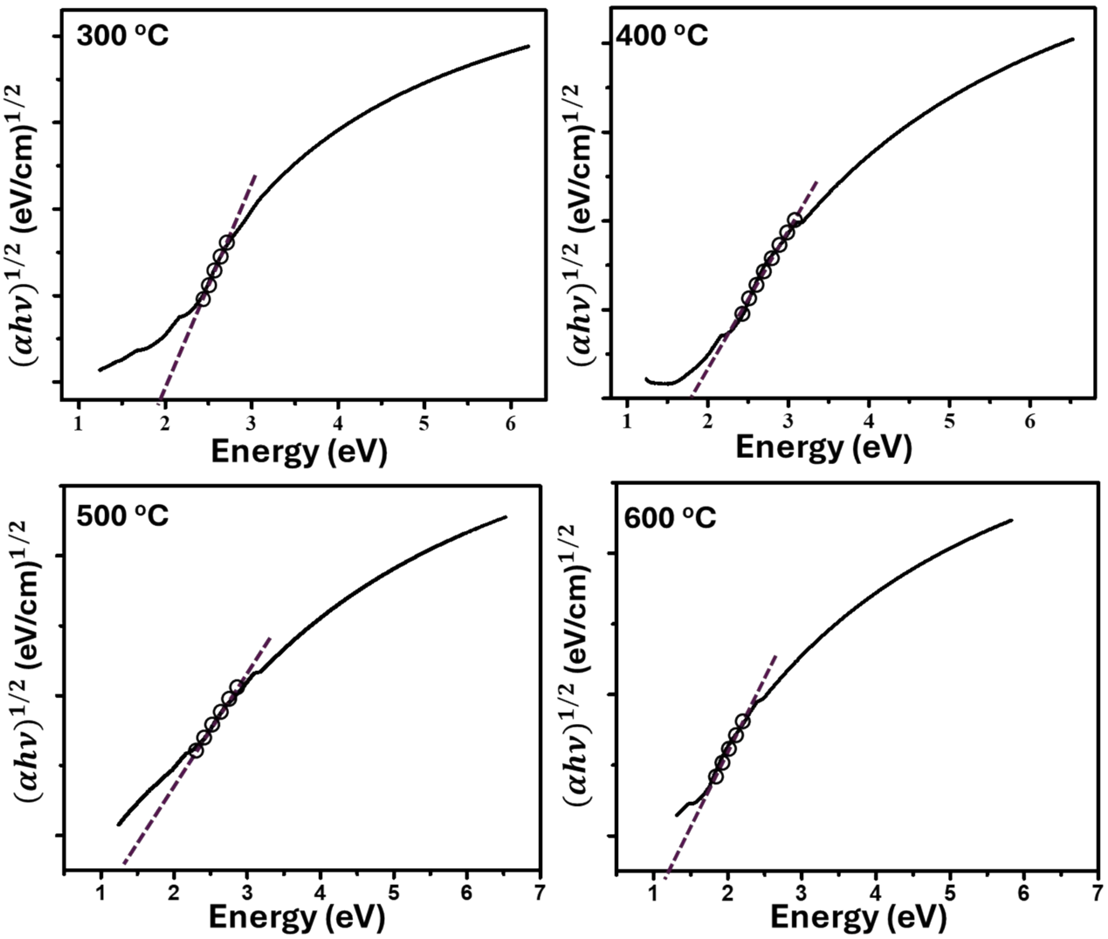

Figure S1. Tauc plots from UV-Vis absorption spectra for SnSe thin films synthesized at different growth temperatures: 300 °C, 400 °C, 500 °C, and 600 °C. The extracted optical band gaps decrease from 1.9 eV (300 °C) to 1.8 eV (400 °C), 1.4 eV (500 °C), and 1.2 eV (600 °C), indicating a temperature-dependent band-gap narrowing associated with improved phase purity

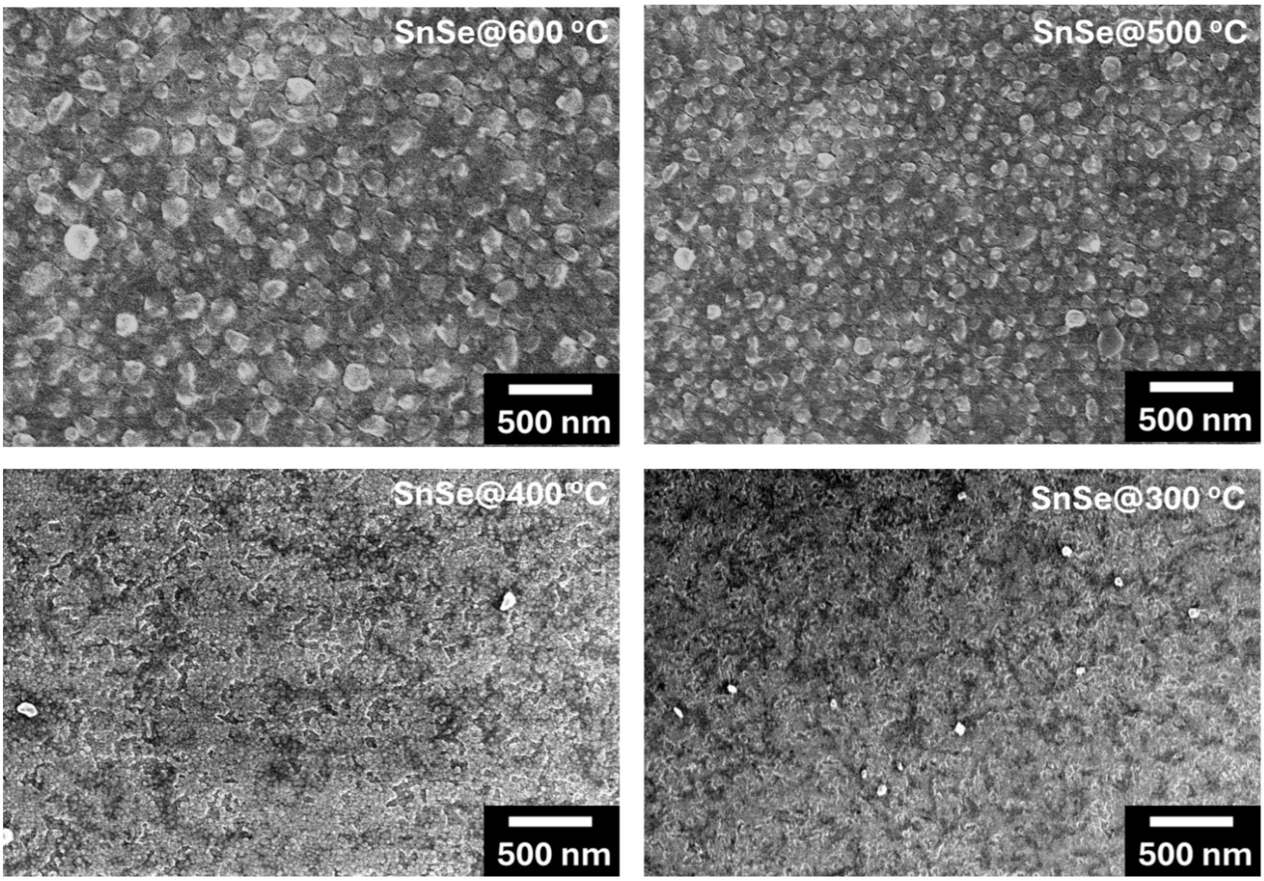

Figure S2. FESEM micrographs of SnSe thin films synthesized at different growth temperatures: (a) 300 °C, (b) 400 °C, (c) 500 °C, and (d) 600 °C. A clear temperature-dependent evolution in surface morphology is observed, with an increase in grain size at higher synthesis temperatures. The scale bar in all images corresponds to 500 nm.
